# On Anæsthesia

**Published:** 1864-05

**Authors:** 


### Selections.

ON ANÆSTHESIA.—*Anæsthesia; Wm. T. G. Morton and his knowledge of the discovery of Wells; of its verification by experiment, and of its introduction into dental and surgical practice at Hartford.*

EDITOR MED. AND SURG. REPORTER:—

While the deeply interesting events which I have depicted were transpiring at Hartford, while one of the most astonishing secrets of nature was being detected and dragged to light, and while dentists and surgeons were performing with means alike effective and harmless feats almost miraculous, and the fact was being blazoned forth and made as notorious as that the State House stood on the public square, or that the celebrated Charter-oak was in its vicinage, how was the distinguished Wm. T. G. Morton occupied? Had he no knowledge of such wonderful developments? No suspicion of their occurrence? Had some anæsthetic been applied to paralyze all his powers physical and mental? Or was he wide awake flying about (as he now pretends) after the very thing which Wells had already brought out at Hartford? I am strongly inclined to think that some bird came flying along, and whispered into the Doctor's ear a slight intimation as to the purport and effect of the Wells discovery or possibly the idea was insinuated, while he was in a mesmeric state, into his mind, by some mysterious agency! At any rate he had it, and the question is where it came from? I think I can satisfy every candid reader on this point.

1. In 1841 or 1842 Morton came from the West and settled at Farmington (only nine miles west of Hartford) as a dentist. He became a pupil of Dr. Wells and was accustomed to come into Hartford for instruction, and also to obtain the assistance of Dr. Wells in getting up work. He married his wife at Farmington. Subsequently, as I believe, in 1843, he removed to Boston, and Wells formed a co-partnership with him to practice dentistry in that city, which he (W.) not long after dissolved for reasons no doubt quite satisfactory to himself. Morton therefore was intimately acquainted with Wells, and it is not at all probable that he would be either inattentive or indifferent to events occurring at Hartford, particularly when intimately connected with his own profession.

2. It is admitted on all hands that, Wells toward the close of December, 1844, that is to say in less than twenty days after he made his great discovery, went to Boston for the purpose of making the fact known to the medical and surgical faculty there. Who would he be more likely to call on than his former pupil and late partner, Morton? In the pamphlet published by Dr. Wells in 1847, he makes the following statement. Being a resident of Hartford, Conn., I proceeded to Boston in December of the same year," (meaning December, 1844), "in order to present my discovery to the medical faculty, first making it known to Drs. Warren, Hayward, Jackson, and Morton, the last two of whom expressed themselves in the disbelief that surgical operations could be performed without pain, both admitting that the modus operandi was entirely new to them." Dr. Morton, in his memoir to the French Academy of Arts and Sciences, states that Dr. Wells did make the visit to Boston at the date, and for the purpose alleged by him. That he (M.) assisted him in securing an opportunity to test the correctness of his theory and the validity of his discovery. That Wells extracted for some person a tooth while under the influence of the nitrous oxyd, but "the patient screamed from pain, and the spectators laughed and hissed." But Dr. C. A. Tuft, then a medical student and present, swears that "the patient hallowed some during the operation, but on his return to consciousness, said he felt no pain whatever." * * Dr. Tuft adds, "I regarded the operation at Boston, above described, as successful, and as proving the truth of Dr. Wells' theory." In his pamphlet Dr. Wells states, "I was then invited to extract a tooth for a patient in the medical class, which operation was performed *but not with entire success*, as the gas bag was removed too soon."

Your readers can hardly fail to notice the scrupulous exactitude of Dr. Wells in stating facts, and here I can not deny myself the satisfaction of presenting another testimonial as to his character in this and other respects. Dr. Abel Ball, dentist, of Boston, swears: "I always regarded Dr. Wells as a man of uncommon talents. He was very enthusiastic, possessed a philosophic and inventive mind, *was very conscientious*, and his character was without a blemish as far as I know."

I will only add further in this connection, that the partial failure of Dr. Wells on the occasion alluded to, constitutes the only foundation on which the persistent claims of both Jackson and Morton rest, that the anæsthetic experiments of Wells were a failure, and that he accomplished nothing. It would be extraordinary, indeed, if in the vast multitude of cases in which the nitrous oxyd has been used there were not some of an equivocal character and even some in which it did not take effect at all. I believe, however, that the number both of the one and the other have been much less than have occurred in the use of any other anæsthetic agent. But a truce to these collateral matters, as my object now is to show, by direct and circumstantial evidence, that Morton was at an early date thoroughly informed on the subject of the Wells discovery, and ultimately became convinced of its validity. It is true, he in the first instance, did not believe in it, and in candor I must admit that his incredulity (considering the novelty of the idea and the remarkable character of the result) is not a matter of surprise, much less of reproach. But he knew that Wells was not a visionary theorist, and that he could rely implicitly on his word; hence having had his attention directed to and fixed on the subject, it is not at all probable that he would lose sight of it; nor did he, as will appear by evidence, to be now presented.

3. In the subsequent part of the same pamphlet, Dr. Wells says: "After I had made the discovery I had frequent interviews with him," (meaning Morton), "and he being aware that I had relinquished my professional business in consequence of a protracted indisposition, requested me to instruct him how to prepare the gas which I had been giving so successfully in Hartford, stating that he wished to make a trial of it in Boston. As this interview was in Hartford, I told him to request Dr. Charles T. Jackson (with whom we were both acquainted) " to prepare him some of it, as he was a chemist. Accordingly Dr. Morton went to Dr. Jackson for the gas, who gave him the ether as being attended with less trouble."

Can there be a particle of doubt as to the truth of this statement? Of course Dr. Wells would hardly be disposed to prepare the gas (a bulky article) at Hartford to be used in Boston. He made a reply alike appropriate and sensible—" Go at once to Dr. Charles T. Jackson, he is a chemist, he will prepare the article for you." And Morton went. Accordingly Jackson, knowing that the nitrous oxyd and the vapor of sulphuric ether produced analogous effects on the human system, "gave him the ether as being attended with less trouble." I suppose Dr. Wells made the last statement on information and belief that all the other facts were within his own personal knowledge. Did Dr. Morton have frequent interviews with Dr. Wells on this subject? Did the former request the latter to prepare for him some of the nitrous oxyd for the purpose stated? Did Wells send Morton to Jackson to prepare it for him? I am not aware that he (M.) has ever denied or controverted these statements, and were he to do so, I should believe them none the less. It is admitted that Morton did go to Jackson, but they do not agree as to what transpired, the former claiming that his only object was to pick up a little secreted information, and the latter, that he put him in possession of all the knowledge he ever had on the subject. But they soon burst upon the world as the joint authors of anæsthesia. No wonder that poor Wells, after narrating the above facts, should exclaim (as he does) with some bitterness, "*these are the individuals who claim to be discoverers!!!*"

4. In the deposition of G. Q. Colton (from which I have already made an extract) he says further: "I soon after left Hartford," (meaning soon after he administered the gas to Dr. Wells, December 11, 1844) "and did not hear anything more on the subject till I saw a few weeks subsequent a paragraph going the rounds of the papers announcing that Dr. Wells was extracting teeth without pain, and I stated on several occasions in connection with that paragraph how and when the discovery originated." It seems, then, that Dr. Wells' discovery got into the newspapers and went "the rounds," or in other words obtained universal publicity. If we assume that Dr. Wells partially failed at Boston, here is an enunciation of complete success at Hartford. Would the fact be likely to escape the notice of Wm. T. G. Morton?

5. In the *Boston Medical and Surgical Journal* of June 18, 1845, may be found a communication from Dr. P. W. Ellsworth, "on the modus operandi of medicine," from which I take the following extract: "The nitrous oxyd gas has been used in quite a number of cases by our dentists during the extraction of teeth, and has been found by its excitement perfectly to destroy pain. The patients appear very merry during the operation, and no unpleasant effects follow." More publicity! and that, too, in Boston! and no less than fifteen months before the extraction of Frost's tooth. I strongly suspect that some just ideas touching the reality of the Wells discovery commenced about those days to percolate through his cranium and ere long reached his brain, and became so fixed as to give shape and direction to his future course, whether to his credit or discredit need not be considered here.

6. Mrs. Esther W. Walton, of Sherbrook, Canada East, swears (after speaking of a dental operation which she had had performed at the office of Dr. Morton in January, 1845),— "I was within hearing of a conversation between Dr. Wells and Dr. Morton relative to the discovery of an agent by Dr. Wells whereby he had been and was enabled to extract teeth without occasioning pain. This discovery Dr. Wells communicated to Dr. Morton at this interview," (meaning that he had then made known to him in substance at least, what it was). "In the early part of the conversation, the precise words of which I can not recall, Dr. Morton made light of it, treating the subject as chimerical. This incredulity on the part of Dr. Morton seemed to touch the feelings of Dr. Wells, and induced him to remark, "*I have done it and can do it again.*"

7. Oswin R. Roberts, of Hartford, Conn.—

"Dr. Wm. T. G. Morton called at our office this winter, prior to January 1st, 1853, and had a long conversation with us respecting the discovery of anæsthetic agents." * * "Dr. Morton stated he took his idea from Wells' use of the nitrous oxyd gas, but that the gas failed, and he went on perfecting the discovery until it resulted in the use of sulphuric ether!"

"Took the idea from Wells," good so far! but nitrous oxyd failed! Fortunately the ipse dixit of Wm. T. G. Morton can not settle that question! Went on perfecting the discovery! which was no discovery after all, as it failed! Until he run the whole affair into the inhalation of the vapor of ether! the two substances being in their effects well known equivalents for each other! Verily, Morton must hereafter be recognized as the Columbus of the scientific world!!

8. In conclusion, I will present extracts from two depositions, the first from the deposition of Dr. Grenville G. Hayden, taken and approved by Dr. Morton before the select committee of which Col. Bissell was chairman (First Session, Thirty-Second Congress), and the second from the deposition of Dr. James McIntyre, taken and offered by Dr. Jackson before the select committee of which the Hon. Mr. Edwards was chairman. (Second Session, Thirtieth Congress.)

In the first deposition Dr. Hayden, among other things, testifies as follows:

"That about the last of June, 1846, Dr. Wm. T. G. Morton called upon me at my office No. 23 Tremont Row, and stated to me that he wished to make some arrangements with me that would relieve him from all care as to the superintendance of those employed by him in making teeth and all other matters in his office. He stated as a reason for urging me to superintend his affairs in his office that *he had an idea in his head* connected with dentistry which he thought would be one of the greatest things ever known, and that he wished to perfect it and give his whole time and attention to its development. Being extremely urgent in this matter, I made an engagement with him the same day according to his request. I then asked him what his secret was. "Oh," said he, "you will know in a short time." I still insisted on knowing it, and he finally told me the same night, to wit: the night of the last day of June, 1846, aforesaid, that it was something he had discovered which would enable him to extract teeth without pain. I then asked him if '*it was not what Dr. Wells his former partner had used*,'' and he replied, "*Nothing like it*,' and furthermore that it was something that neither he nor any one else had ever used."

Here is, in substance, an explicit acknowledgment by Dr. Morton, that he, at the date named, was not only well informed as to what anæsthetic agent Wells had been and was using, but also as to its nature and properties, for otherwise how could he declare (as he did peremptorily) that he contemplated introducing another agent *entirely unlike it.*

In the second deposition Dr. McIntyre testifies as follows:

"In the month of September, 1846, I was a student in chemistry with Dr. Charles T. Jackson, of Boston. In the latter part of September I was sitting in the front room or office of Dr. Jackson's laboratory, when Mr. Wm. T. G. Morton came in and asked for Dr. Jackson, and passed through the office into the house adjoining the laboratory. In a short time Morton came into the back room with an India rubber bag in his hands. Dr. Jackson came in with him, or shortly afterwards. Dr. Jackson asked Morton what he wanted with the bag. He said he wanted to blow up the bag, and act upon a patient's imagination by making her breathe from the bag. The precise words of Morton's answer I do not remember, but the purport of it was, that he wanted to extract some teeth from a lady who objected on account of the pain, and that he expected, by making her breathe from the bag, to believe that she would suffer no pain from the extraction of her teeth. * * * *There was then some conversation about the use of exhilerating gas; whether it was first mentioned by Dr. Jackson or Morton I do not remember. Morton asked him if he (M.) could not make it. Dr. Jackson told him that he could not succeed without apparatus and the assistance of some one who had some chemical knowledge, and that if he undertook to make it, he would get nitric oxyd instead of nitrous oxyd. He asked Dr. Jackson if he would not prepare some for him. This Dr. Jackson declined on account of his business.*" * * * * As he was going, Dr. Jackson told him that he could tell him something that would make his patients insensible, and then he could do what he had a mind to with them. Morton asked him what it was. Dr. Jackson then told him to go to Burnett's and get some pure sulphuric ether, and pour it on a handkerchief, and put it to the patient's mouth, and let her inhale it. Morton asked what sulphuric ether was,—what sort of looking stuff it was. I stayed in the front room while Morton and Jackson went to look at the ether. From Morton's questions about the ether, I am satisfied he knew nothing about its properties or nature. I heard Morton ask Jackson very particularly whether it would be safe to use. Dr. Jackson assured him that it was perfectly safe, and alluded to the students at Cambridge having used it. Morton appeared to be afraid to use the ether, and asked him several times if it was safe. Dr. Jackson advised Morton to try it on himself. Morton asked me if I would be willing to try it. I told him I would. * * * The next day after the above conversation Morton came into the office and told Dr. Jackson that the ether had worked nicely—that the patient suffered no pain." In these statements the witness is fully sustained by the testimony of Dr. Geo. O. Barnes, also adduced by Dr. Jackson on the same occasion, and on the facts thus disclosed I submit the following remarks:

1. Morton went to Jackson to obtain the very instrument which Wells had been using for near two years at Hartford, in administering the nitrous oxyd, to wit: an India rubber bag.

2. He carried with him the idea of inhalation, and adverted to it early in the interview. No doubt he looked very grave when he told Dr. Jackson that he was about to inflate the bag with common air to operate on the imagination of a reluctant patient, and not on her nerves of sensation; and, no doubt, he was as frank, sincere, truthful, and honest in making this statement as he usually has been when speaking of anæsthesia, or any subject connected with it.

3. Although common air was on his lips, yet it is quite obvious that the nitrous oxyd and the wonderful success which had attended it, was on his mind. He therefore commenced talking about it. I say, "*he commenced*," notwithstanding, Dr. McIntyre says he does not recollect who introduced the subject. Obviously Dr. Jackson had no occasion to advert to it.

4. It is morally certain that Morton called on Jackson on the occasion named, in conformity with the recommendation of Wells, and for the purpose contemplated by them both at Hartford. Why so much disingeniousness? Had he then conceived the idea of appropriating the Wells discovery to his own use?

5. Dr. Jackson seems to have interrogated him very closely as to the use which he proposed to make of the India rubber bag, and was not quite prepared to swallow his "common air" humbug, and this led to a little cross interrogation by Morton. He wanted to know whether he (Morton) could not prepare the nitrous oxyd, to which Dr. Jackson properly replied in the negative, as he knew nothing about chemistry, and would be in danger of producing the nitr*ic* oxyd (a rank poison) in place of the nitr*ous* oxyd. He then requested Dr. Jackson to prepare some for him, which that gentleman was obliged to decline by reason of other engagements.

6. It is quite apparent that Dr. Morton went to Dr. Jackson's office under an idea that if he could get an India rubber bag, he could contrive in some way to fill it with the nitrous oxyd, and if he could have done so surreptitiously so much the better for his purpose. But finding this impracticable he spoke out as to the fact; he wanted to use the nitrous oxyd to obviate the pain (often exquisite) resulting from the extraction of teeth. At this juncture Dr. Jackson suggested the substitution of the vapor of sulphuric ether as being likely to produce the same effect. This was no discovery on the part of Jackson. Any tyro of chemistry under the same circumstances would have done the same thing.

7. It is certain that this interview between Morton and Jackson must have taken place either on the 29th or the 30th of September, 1846, for Dr. McIntyre says that "the next day after the conversation, Morton came into the office and told Dr. Jackson the ether worked nicely—that the patient suffered no pain." Now Frost's tooth was extracted on the 30th just at dark, and it is not probable that Morton reported the result to Jackson the same evening. If we assume that the report was made on the succeeding day, that is to say, October 1st, then the interview with Jackson must have been on the 30th, so that Morton obtained the information from Jackson (as to the substitution of the vapor of ether for the nitrous oxyd) and the ether itself from Burnett's, and extracted Frost's tooth, all on one and the same day, and this, I am satisfied, was the fact.

8. Whether Morton had any real knowledge at the date specified, of those intoxicating or exhilarating substances, the nitrous oxyd and sulphuric ether, it will not be difficult to infer from the statements of Dr. McIntyre. He seems to have been a complete ignoramus as to both.

In conclusion, I will only say that the many considerations which I have adduced under this head of inquiry, must as I conceive, be abundantly adequate to bring every upright intelligent mind to the conclusion that Morton must at all times, and particularly in the summer and fall of 1846, have been well informed, touching the inception of anæsthesia at Hartford, and its full development there. *He knew that anæsthesia had been brought out! had been made practical! and that the conception of Wells in all its deeply interesting proportions had been revealed to the community in which he resided, and must soon be revealed to the world—a magnificent reality.* A Lover of Truth and Justice.

